# Reversal of a Suspected Paradoxical Reaction to Zopiclone with Flumazenil

**DOI:** 10.1155/2016/3185873

**Published:** 2016-09-08

**Authors:** Zarah Jordahn, Cheme Andersen, Anne Marie Roust Aaberg, Frank Christian Pott

**Affiliations:** ^1^Department of Anaesthesiology, Rigshospitalet, Glostrup Matriklen, Copenhagen, Denmark; ^2^Department of Anaesthesiology, Copenhagen University Hospital, Hvidovre, Denmark; ^3^Department of Anaesthesia and Intensive Care, Bispebjerg Hospital, Copenhagen, Denmark

## Abstract

We describe the care for an elderly woman who was admitted to the intensive care unit (ICU) to receive noninvasive ventilation for acute exacerbation of chronic obstructive pulmonary disease. After administration of the sleeping pill zopiclone, a nonbenzodiazepine receptor agonist (NBRA), the patient became agitated and was confused, a possible paradoxical reaction to benzodiazepines. These symptoms were immediately resolved after treatment with flumazenil, usually used to reverse the adverse effects of benzodiazepines or NBRAs and to reverse paradoxical reactions to benzodiazepines. This case indicates that zopiclone induced behavioral changes resembling a paradoxical reaction to benzodiazepines and these symptoms may be treated with flumazenil.

## 1. Introduction

Delirium is a disturbance of consciousness and cognition that develops within hours or days. It fluctuates and appears in up to 80% of patients in the intensive care setting and has a multifactorial etiology [1]. Approaches to prevent delirium include pharmacological and nonpharmacological interventions [2]. Sleep disturbances are associated with delirium, and while the exact causal relationship is unknown, securing sleep of hospitalized patients is imperative [3, 4]. Thus, between 41% and 96% of elderly patients admitted to the hospital are treated for sleep disturbances [5]. Drugs often used for this purpose are the so-called Z-drugs (zolpidem, zopiclone) also referred to as nonbenzodiazepine receptor agonists (NBRAs). Like benzodiazepine, Z-drugs act on the gamma-aminobutyric acid-A-receptor (GABA-A-receptor) complex, by improving sleep quality and reducing sleep latency [6]. Reports of side effects are rare but may include complex behavior such as sleepwalking and sleep eating, without any recollection of the event [7]. We describe a patient who became agitated and aggressive several hours after administration of zopiclone. This resembled delirium but may have been a paradoxical reaction to zopiclone [8]. The symptoms immediately resolved after treatment with flumazenil, usually used to reverse the adverse effects of benzodiazepines or NBRAs but can also reverse paradoxical reactions to benzodiazepines [9, 10].

## 2. Case Presentation

An 81-year-old woman with severe chronic obstructive pulmonary disease presented to the medical emergency department with dyspnea. She was diagnosed with acute exacerbation of chronic obstructive pulmonary disease caused by pneumonia and was treated with antibiotics, inhaled bronchodilators, systemic corticosteroids, and noninvasive ventilation. When she failed to improve, she was transferred to the intensive care unit, and to treat her sleeping difficulties dexmedetomidine (100 *μ*g/mL) was infused at a rate of 8–14 mL/h, without effect, however. On the following night, one tablet of 7.5 mg zopiclone was given with initially good effect. After four hours of noninterrupted sleep, the patient woke up agitated, confused, and restless. Since these behavioral changes resembled a paradoxical reaction to benzodiazepine, 0.2 mg flumazenil was given intravenously. After a few minutes, all pathological behavioral symptoms resolved and did not reappear during hospitalization. The patient had no recall of the episode. Previous intake of zopiclone was not registered in her medical charts, but it is unknown whether the patient had taken zopiclone before. She had no history of alcohol abuse or intake of sedatives, benzodiazepines, or antipsychotic drugs. With the exception of dexmedetomidine, no other drug suspected to interfere with the metabolism of zopiclone was given.

The patient was transferred from the ICU to the medical ward and after 3 days discharged to a temporary care center. Two days later, exacerbation in her chronic obstructive pulmonary disease resulted in readmission to the hospital, where the decision was taken to cease active care. The patient died shortly after.

## 3. Discussion

This report describes reversal of an assumed paradoxical reaction to zopiclone with flumazenil in an elderly woman. While paradox reactions and complex behavior associated with the administration of benzodiazepines are well described phenomena [8], to our knowledge, only two cases of agitation following Z-drug administration have been published [11, 12]. In contrast, case reports on complex behavior are more abundant [7], and, in these cases, discontinuing the drug resolved the symptoms.

The mechanism behind complex behavior and paradoxical reactions remains unclear. Predisposing factors such as age, sex, concomitant disease states, psychological disturbances, and/or alcohol abuse have been proposed [7, 8]. Benzodiazepine and Z-drugs act on the GABA-A receptor, producing effects such as sedation, sleep induction, and amnesia. The GABA-A-receptor complex consists of several heterogenic subunits; the different subunits and their combination create different subtypes of the receptor. While benzodiazepines have a high affinity for several subunits, Z-drugs mostly bind to the *α*-1-subunit [13].

Z-drug concentrations exceeding the recommended level have been suggested to be responsible for the occurrence of complex behaviors [7]. Whether intentional or accidental or as a consequence of drug-on-drug action, a concentration above the recommended dose may lead to decreased selectivity of Z-drug to the *α*-1-subunit, thus producing effects more like those of benzodiazepines, including paradoxical reactions. Our patient received the recommended dose, but, considering her age and state of health at the time of administration, a relative overdose of the drug could be the cause of the reaction seen. Furthermore, prior to the administration of zopiclone, the patient was treated with dexmedetomidine, which, like zopiclone, is metabolized in the liver [14]. Zopiclone is metabolized by CYP 3A4 and inhibitors of cytochrome oxygenase can increase plasma concentration of this drug and therefore may induce side effects. However, no drugs that inhibit CYP 3A4, like macrolides, were given. Intoxication/overdose was not suspected, but toxicological screening was not performed. We can only speculate as to whether genetic variation in the function of the GABA receptor subunit genes had a role in developing the unexpected effects observed.

Flumazenil acts as an antagonist on the GABA-A receptor and is primarily used as antidote in benzodiazepine intoxication and benzodiazepine-related paradoxical reactions [8, 15, 16]. Effect on Z-drug overdose and reversal of the associated adverse effects has also been reported [9, 17–19] but the exact mechanism of action of flumazenil is unknown. Flumazenil is generally well tolerated but should be used with caution in patients chronically treated with benzodiazepine and where benzodiazepines are given to control epileptic seizures.

Our patient partly fulfilled the criteria for development of delirium. A causal relationship between the use of Z-drugs and development of delirium was not demonstrated. A single test dose of flumazenil may be considered for elderly patients who develop delirium after administration of Z-drugs.
